# Personality and Cognitive Profiles of Animal-Assisted Intervention Dogs and Pet Dogs in an Unsolvable Task

**DOI:** 10.3390/ani11072144

**Published:** 2021-07-20

**Authors:** Patrizia Piotti, Mariangela Albertini, Lidia Pia Trabucco, Lucia Ripari, Christos Karagiannis, Claudio Bandi, Federica Pirrone

**Affiliations:** 1 Department of Veterinary Medicine, University of Milan, Via dell’Università 6, 26900 Lodi, Italy; patrizia.piotti1@unimi.it (P.P.); federica.pirrone@unimi.it (F.P.); 2 Department of Bioscience, University of Milan, Via Celoria 26, 20133 Milano, Italy; lidia.trabucco@studenti.unimi.it (L.P.T.); claudio.bandi@unimi.it (C.B.); 3 Independent Researcher, Località Rifoglieto, 55011 Altopascio, Italy; ripari.lucia@gmail.com; 4 Hellenic Institute of Canine and Feline Behaviour & Training, 10434 Athens, Greece; ckaragiannis@behaviour.gr

**Keywords:** unsolvable task, Animal-Assisted Interventions, referential looking, personality, dog

## Abstract

**Simple Summary:**

Dogs are genetically predisposed to communicate with humans in collaborative ways, and life experience enhances this ability. The role of canine personality on this predisposition is unknown. This study used a test where dogs encountered an unsolvable problem. The aim of the study was to understand whether dog personalities could predict their tendency to look at the owner, approach and engage with the task, and show distress during the task. We also compared dogs with life experience in Animal Assistance Interventions and dogs with no work, training, or sport experience. The results indicate that the dogs with a personality more sensitive to uncertain situations looked at the owner more and engaged less often with the task, suggesting they were expecting directions from the owner. Dogs with a personality more sensitive to obtaining rewards and avoiding punishment looked at the owner less often. Dogs with a high tendency to avoid punishment also abandoned and returned more often to the task, possibly as a sign of frustration. Finally, Animal-Assisted Intervention dogs looked more at the owner and had fewer task orientations, confirming the enhancing effect on looking. As personality differences reflect genetic predispositions, it is very important to know the role of personality on cognitive traits that are important to dogs that work and collaborate with humans.

**Abstract:**

Dogs are biologically predisposed to communicate with humans in cooperative contexts. They show individual differences in dog–human communication and inhibition, potentially enhanced by life experience, e.g., Animal-Assisted Interventions. This study aimed to investigate whether dogs’ personality, defined by biologically meaningful neural circuits described in the Reinforcement Sensitivity Theory of Personality (RST), predicted dogs’ communication, task orientation, emotional state, and approach of an unsolvable task. We also investigated the differences between dogs experienced in Animal-Assisted Interventions (AAI) and inexperienced dogs. The results indicated that a high sensitivity to the RST personality trait related to managing uncertainty (Behavioural Inhibition System, BIS) predicted fewer task orientations but increased referential and non-referential looking, which we interpreted as a way to obtain directions from the owner. Conversely, a high sensitivity to the traits reflecting tendencies to approach rewards (Behavioural Approach System, BAS) and avoid punishment (Fight–Flight–Freeze System, FFFS) predicted lower looking. High sensitivity to the FFFS also predicted more frequent task orientations, which we interpreted as frustration. Finally, the dogs in the AAI program looked more at their owner and were less oriented towards the task. These results provide empirical evidence of individual differences tied to the psychobiological personality traits in canine cognitive skills. Understanding such cognitive profiles may have an enormous impact on activities that rely upon dog–human collaborative interaction.

## 1. Introduction

The unsolvable task is a cognitive test originally designed to compare looking behaviour between domestic dogs and wolves socialised to humans when facing an unsolvable problem [1]. During this task, the animals are initially given access to some food they can retrieve from a container while in the presence of a human partner. After they have learned to retrieve the food, the apparatus is altered so that the food becomes inaccessible; thus the task is unsolvable. In this situation, it was originally observed that the dogs looked back earlier at the humans and spent more time looking at them when compared to the socialised wolves [1]. Additionally, the authors noted some behavioural variability in the wolves [1]. In humans, eye contact is understood as initiation and maintenance of communication [2,3,4,5], therefore the authors of the “unsolvable task” study proposed that, by evolutionary and ontogenetically processes, the readiness of dogs to look at the human face had led to complex forms of dog–human communication that could not be achieved in wolves even after extended socialization [1].

Over the years, researchers have investigated multiple aspects of dogs’ looking back during the unsolvable task. Several authors believe that the latency to look at the human partner during the unsolvable task may be a by-product of dogs’ persistence in interacting with the food container, meaning that dogs look at the human partner after they have exhausted other strategies to try and solve the problem independently [6,7,8]. Or, we may say that dogs will use all the strategies they have learnt to obtain the food in similar contexts, including gazing at humans. 

However, Lazzaroni and colleagues have pointed out that, in the unsolvable task, dogs do not specifically look at humans as a cognitive strategy to solve the problem; rather, they are very prone to look at humans when food is around, due to previous experience in similar contexts [8]. In fact, dogs’ tendency to look at humans is affected by training and experience during the ontogeny of the dog. This may be the case because the owner’s directed attention leads to the improved effectiveness of the owner as a source of instructions on what to do (helpful communication) or in reaching a goal (instrumental help) [9,10,11].

One unique context of collaborative interaction between humans and animals is Animal-Assisted Interventions (AAI). Animal-Assisted Interventions are a heterogeneous group of activities, including the presence of and/or the interaction with human partners [12]. Pirrone and colleagues observed that gaze synchrony (dog and human partner gazing at each other) and joint attention (dog and human partner both gazing at the same target) were expressed during AAI sessions, with joint attention being the most frequent [13]. In addition, dogs involved in AAI have expressed enhanced sustained attention towards their owner but less frequent looks compared to untrained and agility dogs [12]. 

In a training–extinction–retraining method for a gazing task, dogs involved in AAI looked longer at the human partner, compared to inexperienced dogs (gaze frequency was not measured in this task) [14]. Furthermore, during an unsolvable task, dogs involved in AAI looked (non-referential) at a human partner for a longer time compared to inexperienced dogs, and expressed more frequent gaze alternations (non-referential looking frequencies and gaze alternation durations were not measured in this study) [15]. In a different study, Carballo and colleagues scored dogs’ behaviour during a food retrieval task, rather than measuring it directly. Inexperienced dogs were found to use more of what was called a “social strategy” (with positive loadings of looking at human partners, tail wagging, vocalising, and negative loadings of orientation to the task and food pellets eaten) compared to dogs involved in AAI and dogs trained for sports [16]. However, the different measuring system and merging of different variables make it difficult to compare these findings with previous research. 

Nevertheless, in a modified version of the unsolvable task where humans were not present, Rao and colleagues found that the duration of dogs’ manipulation of the food container (persistence in task orientation), their diversity of actions adopted to try and obtain the food (motor diversity), body posture when approaching the container, and latency to interact with the container (contact latency) likely reflect personality traits [7]. Specifically, different problem-solving canine studies describe a proactive personality type characterised by high persistence, high motor diversity, high activity, and low neophobia [7,17]. These characteristics reflect the features of dogs that are sensitive to the activation of the Behavioural Approach System (BAS), a trait of canine personality as defined by the psycho-biologically derived Reinforcement Sensitivity Theory (RST) [18]. 

According to owners’ reports, high BAS dogs also present behaviours related to trainability and responsiveness in the presence of rewards [18] and have more frequent and better social interactions with their owners [19]. On top of the previous cluster of behaviours, Rao and colleagues describe two other different behavioural clusters: insecure body posture when approaching the container (which reflects neophobia in dogs [20]) and latency to approach the container (which the authors found to be independent from the other two clusters). These two perfectly reflect the other two traits that constitute the Reinforcement Sensitivity Theory framework: the Fight–Flight–Freeze System (FFFS) and the Behavioural Inhibition System (BIS) [18]. In dogs, higher FFFS is predictive of more display of physical discomfort and poorer social interactions with their owner as well as high manifestation of passive avoidance [18,19]. On the other hand, the BIS system has an inhibitory role mediating the careful assessment of an ambiguous or conflicting situation [21]. In dogs, high sensitivity to the BIS is predictive of increased expression of physical discomfort (possibly due to a negative bias [22]) [19] and is associated with low impulsivity [18].

In recent years, there has been increasing attention to the role of personality, or individual differences, on the performance in cognitive tests in non-human animals [23,24,25]. Animals that are bold, proactive, keen on exploration, but also aggressive, tend to be more impulsive and persistent [25,26]. Conversely, animals that are shy and less exploratory are sensitive individuals that carefully sample the environment before making a decision, and are particularly sensitive to change, such as loss of rewards [25]. Accordingly, dogs that are sensitive to the BAS should be expected to be more persistent in the unsolvable task, while dogs that are sensitive to the BIS should be expected to have a higher contact latency. As Rao and colleagues pointed out, insecure postures in the task may be reflective of neophobia [7], therefore it may be expected that dogs sensitive to the FFFS will be more likely to display postures reflective of a negative emotional valence. MacLean and colleagues observed that persistence in the looking behaviour forms a cluster separated from other cognitive aspects [24]. Therefore, it may be hypothesised that personality should also reflect individual differences in dogs’ tendency to look at humans during the unsolvable task. It is, however, difficult to predict the direction of this relationship: it may be that a longer duration of gazing at humans is predicted by high BAS due to the tendency to persist in their behavioural strategy, or it may be that a higher frequency of gazing at humans and gaze alternations are predicted by high BIS as an attempt of gathering information on what to do from the owner.

To our knowledge, these hypotheses have never been tested by measuring dogs’ performance in the unsolvable task and their RST personality traits. The aim of this study was, therefore, to measure the role of RST traits on dogs’ looking behaviour, persistence, contact latency, and neophobia during the unsolvable task. Given the abundance of evidence that being involved in Animal-Assisted Interventions enhances dogs’ attention to humans, we compared two different populations of dogs: a group of dogs that have extensive experience in AAI and a group of untrained dogs. We expected dogs involved in AAI to be more persistent in their looks at humans.

## 2. Materials and Methods

The study was approved by the Animal Welfare Organisation (OPBA) of the University of Milan (OPBA_106_2018). Participants were given written information about the aim and the procedures of the study and the right to withdraw at any time. Before data collection, informed consent was obtained from each participant. Participation was voluntary.

## 2.1. Subjects

A total of 61 dogs (*n* = 27 involved in AAI, *n* = 34 controls) were recruited for this study via opportunistic sampling, by sharing an ad via social media and the word of mouth. For the dogs involved in AAI, the owner had to be registered in the National Registry of the Italian National Reference Centre for AAI (NRC AAI) (www.digitalpet.it, accessed on 15 June 2020), the dog had to have at least one year of experience in AAI (pre-COVID-19 lockdown). They should have taken part in at least 10 sessions. We did not require the dogs to have received any formal training specific for AAI, as this is not required by the Italian regulation. For the control dogs, the criteria were that neither the dogs nor the owners had ever been involved in AAI, and the dogs should have had never taken part with the owner in any training activities, sports, or other forms of training, either as formal classes or informally. All owners had to be above 18 years of age and they should have been living with their dog for at least one year. Following testing, 16 dogs were excluded from subsequent analysis due to technical problems (10 dogs were able to remove or open the food container, one due the owner’s mistake, five because the technical problems with the video recording). The final sample consisted of 45 dogs. The AAI group included 20 dogs (Mdn_Age_ = 5 years, range = 1–8 years, F:M = 2:1), the control group included 25 dogs (Mdn_Age_ = 5 years, range = 2–8 years, F:M = 1:1). The characteristics of the dogs are reported in Appendix A, Table A1.

## 2.2. Material and Protocols

### 2.2.1. Dog Reinforcement Sensitivity Theory Questionnaire

The Dog Reinforcement Sensitivity Theory Questionnaire (Dog-RSTPQ) [18] was recently validated in Italian [19]. The questionnaire consisted of 21 items on a five-point Likert scale, which described the three RST domains, BAS, BIS, and FFFS. A high score indicates high sensitivity to that domain, while a low score indicates low sensitivity. Owners who had agreed to take part in the cognitive test were given a link to fill the questionnaire online and provide their informed consent. Responses to the questionnaire were matched to the video-recordings of test by using the dog’s name, breed, and colour.

### 2.2.2. Unsolvable Test

The test was performed as citizen science, i.e., owners performed the test at home with their dog. To ensure standardisation of the procedure, owners where given written instructions on the material required before taking part in the test: a small Tupperware container that could be firmly shut with a lid, strong tape and double-sided tape, and the dog’s favourite treats. Then, the test was performed during a video-call (via Skype or Microsoft Teams) with one of the researchers (LPT) and the computer or tablet used by the owner for the video call acted as the video camera. After receiving verbal consent, the researcher switched off her camera and started recording the session.

**Figure 1:**
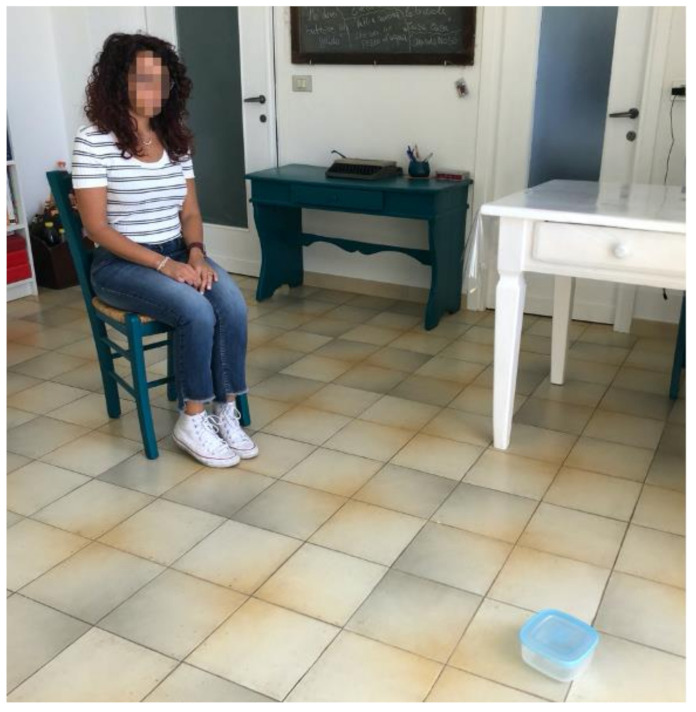
The picture shows the setup for the experiment (the person’s face has been obscured to guarantee anonymity). The owner was sitting on a chair with the container in front of them and the camera was positioned so that both the container and the owner were visible.

The unsolvable task apparatus was similar to that used by D’Aniello and colleagues [11]. The owner placed a chair in a spacious room, ensuring there were no other objects on the floor. They then secured the container on the floor in front of the chair using tape and double-sided tape. The container was positioned about 1.5 m away from the chair, so that the dog was still close to the owner when investigating the container but could not reach it when tethered to the chair with a short leash (Figure 1).

- Solvable trials. Once the video recording had started, the researcher instructed the owner to tether their dog next to the chair, take a piece of food and place it in the container, ensuring that the dog was watching. They placed the lid half-way on top of the container, so that the dog had to touch it and move it away to get the food. The owner then unleashed the dog and sat on the chair while the dog was free to move in the room. During this time the researcher was silent while checking her stopwatch and the owner could look at the dog but was instructed not to gesture or talk at them. The trial ended as soon as the dog ate the food or when 30 s had lapsed. If the dog had not eaten the food, after 30 s, the owner was instructed to help the dog getting it. The trial was repeated three times.
- Unsolvable trial. The unsolvable trial was performed right after the solvable trials with no interruption in between. The trial was identical to the solvable trials; however, this time the owner was instructed to place the lid over the container and close it firmly. Since the dogs had previously experienced that food was accessible from the apparatus (solvable trials), they initially tried to open the container by moving the lid. Upon realising it was now inaccessible, the dogs were expected to engage in other behaviours, including towards the owner (e.g., gaze alternations between the human and the food). The trial lasted for 2 min, then the owner got up from the chair, opened the lid and let the dog have the food.

## 2.3. Video Coding

Digital video footage was taken for all trials and the Solomon Coder software (beta 19.08.02, copyright 2019 by András Péter, developed at ELTE TTK Department of Ethology, Budapest, Hungary) was used to code dogs’ behaviour during testing.

The dogs’ behaviour was coded during the unsolvable trial from the moment the handler released the dog from the lead and concluded when the owner got up to open the container. The dogs’ looking behaviour was recorded based on the orientation of the head and/or eyes of the dog. Smith and Litchfield have argued that inconsistent definitions of the different types of looking have led to discrepancies in the literature [6]. Moreover, looking behaviour should be considered a true communicative and referential gesture if a minimum length is defined [6]. There should be a distinction between referential gazing, defined as a two-step sequence (i.e., target–person or person–target), and gaze alternation, a three-step sequence of alternating gazes between a target and a human partner, within a short period of time—typically 2 s [9,27,28]. Finally, Mongillo and colleagues have argued that when measuring looking behaviour in dogs, frequencies and durations of gazes should be considered as functionally separated [12,29]. Therefore, we coded the following behaviours:

- Looking overall (duration and frequency): Looking was recorded every time the dog turned and lifted their head and/or eyes towards the owner.
- Referential looking (duration and frequency): We followed the definition by Merola and colleagues [27], i.e., a two-way sequence between food and the owner (and vice versa). Only unbroken looks lasting at least 0.2 s were recorded and a gap of no longer than 2 s from the end of each look and the beginning of the following one was allowed, as suggested by Gaunet and Deputte [28] and Marshall-Pescini and colleagues [9].
- Approach latency (latency): The time lapsed from the moment the dog was released to the moment they got in contact with the container. A binary variable recording whether the dog did or did not go to the container was also coded.
- Motor diversity (count): The number of different behaviours that the dog used while interacting with the container. The behaviours observed in the sample were: biting the container, pawing it, sniffing it, holding it with a paw, pawing while biting, pawing while sniffing.
- Task orientation (frequency and duration): The frequency of manipulations and the overall time spent interacting with the container (persistence).
- Negative valence (duration): The time spent displaying postures expressing a negative emotional state, i.e., tail between the legs, tensed body, cautious or jumpy, ears backwards, nose-lick, yawning.

## 2.4. Statistical Analysis

Analyses were performed using R statistical software [30]. The packages lme4 [31], emmeans [32], rcompanion [33], coxphw [34], and survival [35] were used.

A coder blind to the goal of the study and the conditions (LR) coded the referential looking and approach latency, as well as 20% of the video material for the looking overall, motor diversity, task orientation and valence. The main coder (LPT) coded 20% of the video material for the referential looking and approach latency. Coding from the two coders was then compared using Spearman correlations to assess inter-rater reliability.

The personality traits of the Dog-RSTPQ were calculated as described in the literature [18]. The RST-FFFS domain was calculated as the average of questions 1–7 of the RST questionnaire, RST-BIS was the average of questions 8–14, RST-BAS was the average of the questions 15–21 [18]. There were no reversed scores. In addition, dogs were categorised based on whether they received a higher or lower score than the middle score 2.5–3.5). Subsequently, data were analysed using a series of regressions.

Durations were analysed using Linear Regression models (LM), while count and frequency data were analysed using General Linear models (GLM). All models were calculated with the group (AAI vs. control) and the three RST personality traits (BAS, BIS, FFFS) as fixed factors with no interaction; post-hoc comparisons with Tukey correction were then obtained for the group variable. The explained variance of the model was assessed through adjusted R^2^ calculation for continuous response variables, and pseudo R^2^ (Nagelkerke) for count and frequency variables. Distribution of estimates of linear models was visually inspected. Contact latency was analysed using a Cox regression model with the group and RST-BIS categorised trait (high BIS, low BIS, average BIS) as main factors. All dogs scored zero in the negative valence variable, so this could not be further analysed. Significance level was set at α = 0.05.

## 3. Results

All dogs reached the container and ate the food during the solvable trials. During the unsolvable trial, two dogs did not approach the container. Inter-rater reliability was confirmed for all variables (Appendix A, Table A2).

### 3.1. Looking

The GLM for the frequency of overall looking explained 29% of the variance (AIC = 259.29, χ^2^ = 15.17, *p* = 0.004; Table 1). The dogs gazed more often at their owner when they had higher BIS sensitivity (β_BIS_ = 0.13 ± 0.06, *p* = 0.025) and they looked fewer times when they had higher BAS and FFFS sensitivity (β_BAS_ = −0.20 ± 0.08, *p* = 0.011; β_FFFS_ = −0.25 ± 0.09, *p* = 0.007).

**Figure 2:**
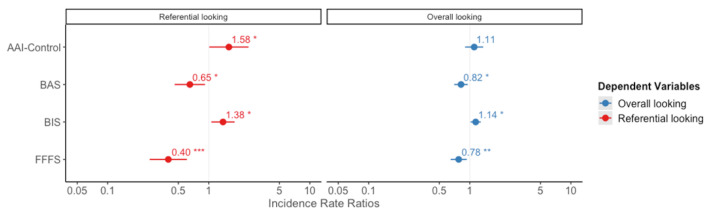
Graph of the LMs for the frequency of referential and overall looking. The dots and numbers represent the Odd Ratios (Expβ) for each fixed factor; the horizontal lines are the standard errors. * *p* < 0.05, ** *p* < 0.01, *** *p* < 0.001. On the right, in blue, is the graph for the overall looking; on the left, in red, is the graph for referential looking.

The LM calculated with the duration of overall looking as response variable explained 32% of the variance (AIC = 291.39, F = 4.79, *p* = 0.003; Table 1). A significant effect of the dog’s group (Control vs. AAI) and the BAS trait was observed. Post-hoc analysis indicated that the duration of looking decreased with increasing BAS sensitivity (β_BAS_ = −21.84 ± 5.59, *p* < 0.001) and that AAI dogs looked longer than the controls (β_AAI-CONTROL_ = 17.90 ± 7.45, *p* = 0.021, Figure 2).

The GLM calculated with the frequency of referential looking as response variable explained 47% of the variance (Table 1). A significant effect of the dog’s group (Control vs. AAI) and the three personality domains included in the analysis (BAS, BIS, and FFFS) was observed (AIC= 146.35, χ^2^ = 27.56, *p* < 0.001). Post-hoc analysis indicated that the dogs involved in AAI (β_AAI-CONTROL_ = 0.46 ± 0.23, *p* = 0.045) and the dogs with higher BIS sensitivity (β_BIS_ = 0.32 ± 0.13, *p* = 0.017) looked more at their owner. Conversely, higher BAS and FFFS sensitivity was predictive of fewer looks at the owner (β_BAS_ = −0.43 ± 0.18, *p* = 0.015; β_FFFS_ = −0.92 ± 0.22, *p* < 0.001).

The LM calculated with the duration of referential looking as response variable explained 31% of the variance (AIC= 252.41, F = 4.56, *p* = 0.004, Table 1). The dog’s group, BAS and FFFS were found to have a significant effect. Specifically, the dogs involved in AAI were much more persistent in looking at their owners compared to the controls (β_AAI-CONTROL_ = 12.20 ± 4.83, *p* = 0.016). Similar to what occurred in the frequencies, higher BAS and FFFS sensitivity were predictive of shorter looks at the owner (β_BAS_ = −9.67 ± 3.62, *p* = 0.011; β_FFFS_ = −11.33 ± 4.28, *p* = 0.011, Figure 2).

### 3.2. Task Orientations, Approach Latency, Motor Diversity

The GLM calculated with the frequency of task orientations events as a response variable explained 44% of the variance (Table 1). A significant effect on the dogs’ group (Control vs. AAI) and the personality traits BIS and FFFS was observed (AIC= 538.84, χ^2^ = 26.18, *p* < 0.001). Post-hoc analysis indicated that the dogs with higher FFFS sensitivity (β_FFFS_ = 0.21 ± 0.09, *p* = 0.030) interacted with the container more frequently. Conversely, the experience in AAI (β_AAI-CONTROL_ = −0.38 ± 0.11, *p* < 0.001) and higher BIS sensitivity were predictive of fewer task orientations (β_BAS_ = −0.43 ± 0.18, *p* = 0.015; β_FFFS_ = −0.92 ± 0.22, *p* < 0.001). However, the LM calculated with the overall duration of task orientation as response variable did not explain the variance better than a null model (AIC= 252.41, χ^2^ = −523.79, *p* = 0.814).

The Cox proportional hazard model (Appendix A, Table A3) indicated that, during the unsolvable task, the time taken to approach the container did not significantly differ between control and AAI dogs or across BIS categories (Wald test = 1.11, df = 3, *p* = 0.800). Likewise, the GLM calculated with motor diversity as response variable did not explain the variance better than a null model (AIC= 114.33, χ^2^ = 5.83, *p* = 0.212).

## 4. Discussion

This study investigated whether dogs’ personality, defined by biologically meaningful neural circuits described in the Reinforcement Sensitivity Theory of Personality, was a good predictor of dogs’ communication, persistence in task orientation, manifestation of negative emotional states, and task approach. The study was also set out to replicate previous findings suggesting that looking at humans is enhanced in dogs involved in AAI, compared to inexperienced dogs. The research paradigm was the unsolvable task [1] performed in a citizen science modality.

Our results indicate that the dogs’ personality traits successfully predicted their looking behaviour towards the owner when facing a problem (unsolvable trial), as well as the number of times they were oriented towards the task. Specifically, the dogs which were more sensitive to the Behavioural Inhibition System (BIS) were found to look more often at their owner, both referentially and non-referentially [6]. Conversely, the dogs which were more sensitive to the Behavioural Approach System (BAS) and to the Fight–Flight–Freeze System (FFFS) were found to look at their owner less often and for shorter periods. The analysis of the frequency of task orientations yielded opposite results, with sensitivity to BIS being predictive of reduced frequency of task orientations and sensitivity to FFFS predicting increased frequency. The dogs’ involvement in Animal-Assisted Interventions was also a good predictor of looking behaviour and frequency of task orientations. When compared to the untrained dogs, the dogs involved in AAI looked both referentially and non-referentially at their owner more often and for longer periods, while they tried less often to interact with the unsolvable task. Finally, none of the variables examined affected approach latency or motor diversity, and there was no expression of negative emotional valence in the sample.

Our findings suggest that the RST personality traits are mapped out in two distinct directions. The BIS was associated with increased communication and decreased task orientation, while the BAS and the FFFS were mostly associated with reduced communication and increased orientation towards the task.

One possible explanation for our findings is that the dogs that are more sensitive to the BIS may be more prone to seek for directions from humans. Referential gazing and gaze alternation are considered examples of referential (i.e., about something) and intentional forms of canine communication [28,36,37]. In the unsolvable task, the looking behaviour has been interpreted by several authors as a help request [6,15]. However, evidence of dogs’ preferential gazing towards better helping-partners is lacking [11,29,38] and even evidence of discrimination between an unfamiliar partner and the owner is contradictory [9,11,15]. This suggests that there may be little cognitive flexibility when it comes to helpful communication in dogs [29]. The most parsimonious explanation is that dogs look at humans during uncertainty to obtain directives [39,40,41,42] or as a request (“look at what I want!”) [42,43,44]. 

According to the Reinforcement Sensitivity Theory, the BIS is activated when an individual is in ambiguous situations, or is facing conflicting goals, and needs to decide what to do: the BIS inhibits the BAS and the FFFS, and activates a cautious assessment of the circumstances [21]. The neural basis of the BIS is the limbic system and, in humans, high sensitivity to the BIS results in individuals that are very careful in evaluating their options, rethinking them over and over to the point that it may lead to anxiety, depression, or compulsive disorders [21,45,46]. In the behavioural ecology and personality fields, cautious assessment and low persistence have been attributed to a “reactive” personality type [25,47], which is reflective of the activation of the BIS. Taken together, these pieces of evidence suggest that dogs that are more sensitive to the BIS may also be more prone to seek directions from humans. 

Furthermore, the reactive personality type is sensitive to change [25], which corroborates the idea that increasing looks at the owners in the high BIS dogs corresponds to seeking indications or to imperative communication, i.e., requests, for the owner when the task outcome changes from solvable to unsolvable. Another explanation could be that dogs which are sensitive to the BIS are simply more active and therefore, they look at their owner more. However, if this was the case, task orientations should also increase. Instead, task orientation followed the opposite direction compared to looking behaviour. In addition, the BIS has an inhibitory role on all behaviours other than cautious assessment [21,48], which would be consistent with the dogs looking at their owner as a means to gather information on the situation, as dogs do [27].

In relation to the BAS and the FFFS, it is possible that frequency of task orientation increased with increasing BAS and FFFS sensitivity as attempts to try and obtain the food alternated with frustration and/or attempts to avoid a negative outcome (lack of reward). In fact, the BAS and FFFS are described in the Reinforcement Sensitivity Theory as two opposite systems, mediating the approach of appetitive stimuli and the avoidance of aversive stimuli respectively [45,48]. The BAS reflects the sensitivity to the activation of the dopaminergic reward circuits in the brain [49], resulting in individuals who are impulsive and very motivated to seek and obtain rewards [21]. The FFFS involves the activation of the amygdala and periaqueductal grey [45,50] and high sensitivity to the FFFS results in individuals prone to interpret novel stimuli as dangerous, or anyway punishing, and avoid them through fearful and aggressive responses or by freezing [21], depending on the perceived intensity of the threat and a chance to escape [18,51]. The FFFS is also activated by the negative experience associated with the lack of an expected reward, i.e., frustration [45]. 

Together, the BAS and the FFFS reflect the behavioural ecology “proactive” personality type, which is persistent, active, exploratory, aggressive, and impulsive [25,47]. It is difficult to explain why personality did not affect the dogs’ persistence in this study in terms of overall time spent on the task, but the number of times they were willing to return to it. It may be that more factors come into play during the task, as the dog looks at the owner and tries other coping strategies. 

In terms of genetic differences, one aspect that should be further investigated when looking at cognitive traits is breed differences. Evidence from the literature clearly indicate that different breeds and breed types vary in their cognitive tendencies. For example, a citizen science project showed an effect of breed-average brain weight on cognitive tasks relying of executive function (memory, pointing, physical reasoning), but not eye contact [52]. Moreover, in a study with portraits of human faces, it was observed that brachycephalic dogs and mixed breeds spent more time looking at the portraits [53]. It was not possible to make inferences regarding breed differences in the current study as this would require a counterbalanced and larger sample size. However, within and between breed differences in cognitive traits should be investigated with a systematic approach.

The lack of relationship between the RST variables and approach latency was somewhat surprising. However, it could be explained by the very low variance within the latency measure. Almost all dogs had very low latencies and only two did not approach the task at all, suggesting a ceiling effect. Furthermore, we observed no signs of negative valence in the dogs’ posture (i.e., tail between the legs, tensed body, jumpiness, ears backward, nose-lick, yawning); thus, the dogs were not likely to be in a motivational conflict at the time they had approached the container in the unsolvable trial. The apparent lack of neophobia may be explained by the differences between our study and that described by Rao and colleagues [7]. In their study, the dogs and the wolves encountered novel objects they had never seen before, while our dogs had likely seen similar containers in the past to those used in the study. Moreover, our dogs lived in families and were tested at home in the presence of a member of their social group (owner), while the canines in the study by Rao and colleagues, although used to being tested and to the testing area, were separated from their group during testing. In addition, our dogs are very likely to have been affected by sampling bias, as it is bound to happen in any cognitive study involving pet dogs, because owners that may believe their dog is likely to enjoy the test are more likely to volunteer. 

It may also be possible that latency measures were not particularly effective as this was a citizen science work: distances varied slightly between dogs and dogs were leashed and unleashed by the owner, rather than an experimenter. While citizen science research is highly effective in investigating various cognitive aspects, such as communication, decision-making, and problem-solving [52,54], it may not be as effective in measuring speed and, therefore latencies. Body expressions of uncertainty or negative valence may be more effective measures. However, when looking at postures and facial expressions, it is important to operate objectively. For example, in this study it was very difficult to operatively define low or insecure body postures, as similar definitions may apply to various degrees of intensity. Objective coding schemes, such as the Dog-FACS (Facial Action Coding Scheme [55]) may be more effective in these circumstances.

The final relationship that we observed was that of experience in Animal-Assisted Interventions with looking and task orientation. Our results indicate that the dogs involved in AAI looked more often and longer at their owners. These findings are consistent with several other studies in the literature suggesting increased use of gaze towards humans in dogs that are involved in AAI [12,14,15]. Mongillo and colleagues suggested that experience in AAI enhances gazing behaviour towards the owner in dogs [12]. Moreover, the coordination of non-verbal behaviour (including gazes) between interactive partners (social synchrony) is associated with empathic and collaborative interactions in mammalian species [56], including dog–human interactions during Animal-Assisted Interventions [13,57]. Animal-Assisted Interventions are likely to be occasions of high ambiguity due to the spontaneous nature of the interaction with vulnerable people. It may therefore be that repeated experience enhances dogs’ ability to seek for indications from their owners.

Social referencing could be influenced by other aspects unrelated with the dogs’ experience in AAI. To control for this, we required a minimum length of the ownership of at least one year for all participants. We requested the control dogs not to have any other experience (training and otherwise) that could have influenced their tendency to look at their owner. It is possible that some owners of the control dogs had involuntarily or indirectly reinforced their dog for attention or gazing. Since personality is both affected by genetics and experience [58], we believe these random influences would be captured by the analysis of individual differences of this study. Systematic, longitudinal research is necessary to tease apart the genetic and the social aspects of personality and cognitive traits. 

Another non-exclusive hypothesis raised by our findings is that dogs that become successful in AAI may have a personality predisposition for this enhancing effect. A genetic predisposition for communication with humans has been established in dogs [59]. Some may argue that breed differences could explain the variation in the dogs’ gazing at human faces in our sample. As breed analysis was not the primary goal of this research, all breeds were accepted in this study, so the sample would be as representative as possible of the real population of pet dogs and dogs involve in AAI in Italy. Unsurprisingly, in our sample, mesocephalic dogs and breeds selected for distant communication with humans, such as retriever types, were overrepresented in the AAI group, while the control group had a larger number of brachycephalic dogs. 

Due to the low number of dogs for each breed, it was not possible to perform statistical analyses to investigate the role of breeds in this sample. In addition, information about the genealogy of the dogs (e.g., registration to a kennel club) was not available; thus, it was not possible to know if these were purebred dogs or simply similar to a certain breed type. Nevertheless, a recent study suggests that mesocephalic dogs look at images of human faces for longer, compared to brachycephalic dogs [53]. However, large-scale research indicated no differences in breed averages for dog–human eye contact [52]. It is therefore not yet clear how breed and face anatomy may affect dog gazing. It is possible that, in addition to breed differences, experience in AAI enhances already existing cognitive traits in dogs. Large-scale research within and between breeds is needed to further understand this possibility.

One issue with AAI is that it comprises extremely heterogenic activities, types of associations, professional figures, and animals’ preparation. However, within this field, Italy offers a unique example of standardisation [60]. In 2009, the Italian National Reference Centre for AAI (NRC AAI) was created, followed in 2015 by the release of the National Guidelines for AAI, which have become operative at national level in 2019 [60]. The Italian National Guidelines define AAIs as interventions with therapeutic, rehabilitation, educational and recreational goals, with the involvement of pet animals [61]. The guidelines standardise the training of the professional figures that need to be involved in the interventions. They also describe the health, behavioural, and training requirements for the animals involved, requiring that any form of training should nurture the human–animal bond and the domestic animals’ spontaneous tendency to form a relationship with humans [61]. The Italian guidelines thus create and ideal opportunity for large-scale research within the field of AAI.

## 5. Conclusions

Overall, the results of our study suggest that biologically defined personality traits, describing tendencies to approach, avoid, and manage conflicting motivations, are predictive of dogs’ communicative behaviour and task orientation within a complex situation. Our findings confirm the growing evidence in the literature that there are cognitive differences between individuals, or cognitive profiles [17,24,25]. Cognitive profiles for communication factors and inhibitory control are stable over dogs’ lifespan and highly heritable [54,62]. Dogs are required to use their cognitive abilities every time they need to decide how to behave and respond to everyday life events. However, cognition plays a particularly important role in working dogs. The work of dogs for the blind, detection dogs, and search-and-rescue dogs relies enormously on dog–human communication; assistance and therapy dogs, police and military dogs must continuously exert inhibitory control during their work. Therefore, better understanding of canine cognitive phenotypes and the role of human selection as well as experience, may have an enormous impact on all activities that rely on dog–human collaborative work.

The authors believe that dogs are biologically predisposed [63] to communicate with humans in cooperative contexts due to domestication and human selective pressure in a wide range of tasks requiring dog–human communication [1,39,41,64,65,66]. What remains unclear is the domestication process that impacts dogs’ inhibitory skills [67,68,69]. Nevertheless, the neural bases for inhibitory control in dogs are well known [70,71]. Psychobiological frameworks [21,58,72,73], being strongly tied to the fundamental biological processes of approaching resources, avoiding threats, and managing motivational conflict, are particularly useful in helping the understanding of cognitive and evolutionary mechanisms. Evidence suggests that cognitive science needs to pay more attention to individual differences within a population, together with comparisons at the population level. We propose that approaches such as the Reinforcement Sensitivity Theory of Personality may be particularly useful to this process.

## Figures and Tables

**Table 1:** Results of the regressions on the looking behaviour during the unsolvable task. Model estimates, (SE), *p* value.

Predictors	Overall Look (Frequency)	Overall Look (Duration)	Referential Looking (Frequency)	Referential Looking (Duration)	Task Orientations (Frequency)
	R_p_^2^ = 0.29, *p* = 0.004	R^2^ = 0.32, *p* = 0.001	R_p_^2^ = 0.47, *p* < 0.001	R^2^ = 0.31, *p* = 0.001	R_p_^2^ = 0.44, *p <* 0.001
AAI vs. Control	0.10 (0.10) 0.342	17.90 (7.45) 0.021	0.46 (0.23) 0.045	12.20 (4.83) 0.016	−0.38 (0.11) <0.001
BAS	−0.20 (0.08) 0.011	−21.84 (5.59) <0.001	−0.43 (0.18) 0.015	−9.67 (3.62) 0.011	−0.06 (0.08) 0.404
BIS	0.13 (0.06) 0.025	−2.06 (4.10) 0.618	0.32 (0.13) 0.017	3.25 (2.66) 0.228	−0.18 (0.06) 0.002
FFFS	−0.25 (0.09) 0.007	−4.55 (6.59) 0.494	−0.92 (0.22) < 0.001	−11.33 (4.28) 0.011	0.21 (0.09) 0.030

Note: FFFS: Flight–Fight–Freeze System. BIS: Behavioural Inhibition System. BAS: Behavioural Approach System. Grey highlight: *p* < 0.05.

## Data Availability

Raw data are not publicly available due to data protection policy; they can be obtained upon request from the corresponding author.

## Appendix A

**Table A1:** Demographic data of the dogs.

ID	Age (years)	Sex	Breed	Colour	Group	Years in AAI
1	6	Female (intact)	Retriever	Yellow	AAI	4.5
2	3	Female (neutered)	Retriever	Champagne	AAI	2
3	5	Male (neutered)	Retriever	Golden blonde	AAI	5
4	5	Male (intact)	Swiss Shepherd	White	AAI	3
5	4	Male (intact)	Beauceron	Black & White	AAI	3
6	4	Female (neutered)	Bernese Mountain Dog	Tri-coloured	AAI	2.5
7	4	Male (intact)	Mixed breed	Black & White	AAI	3
8	6	Female (intact)	Retriever	Yellow	AAI	4
9	7	Female (neutered)	Mixed breed	Beige	AAI	2
10	7	Female (neutered)	Retriever	Golden	AAI	4
11	7	Female (intact)	American Staffordhire Terrier	Brindle	AAI	5
12	6	Male (neutered)	Standard Poodle	Black	AAI	5
13	2	Female (neutered)	Retriever	Champagne	AAI	1
14	5	Male (intact)	Mixed breed	Brown & White	AAI	2
15	2	Male (intact)	Retriever	Champagne	AAI	1
16	8	Female (neutered)	Retriever	Yellow	AAI	7.5
17	6	Female (neutered)	Belgian Sheperd Malinois	Brown	AAI	2
18	2	Female (neutered)	Retriever	Yellow	AAI	1
19	6	Female (intact)	Retriever	Black	AAI	3
20	4	Female (neutered)	Retriever	Yellow	AAI	1
21	3	Female (neutered)	Short-haired Hungarian Hound	Brown	AAI	1
22	3	Female (neutered)	English Cocker Spaniel	Black & tan	AAI	2
23	4	Male (intact)	Retriever	Yellow	AAI	2
24	4	Male (intact)	Retriever	Black	AAI	3
25	6	Male (intact)	English Setter	White & orange	AAI	2
26	6	Female (neutered)	Argentine Dogo	White	Control	-
27	8	Male (intact)	Mixed breed	Black & tan	Control	-
28	3	Female (intact)	Standard Poodle	Champagne	Control	-
29	6	Female (intact)	English Cocker Spaniel	Black	Control	-
30	4	Male (intact)	Pitbull	Black	Control	-
31	7	Male (intact)	West Highland White Terrier	White	Control	-
32	3	Male (intact)	Beagle	Three-coloured	Control	-
33	7	Female (neutered)	West Highland White Terrier	White	Control	-
34	3	Male (intact)	French Bulldog	Black & White	Control	-
35	1	Male (intact)	Standard Poodle	Black	Control	-
36	2	Male (intact)	French Bulldog	Blue & tan	Control	-
37	4	Male (intact)	Chow Chow	Brown	Control	-
38	1	Male (intact)	Dachshund	Black & tan	Control	-
39	4	Female (neutered)	Mixed breed	Brown	Control	-
40	5	Female (neutered)	Mixed breed	Black & White	Control	-
41	6	Female (neutered)	Retriever	Black	Control	-
42	8	Female (intact)	Chihuahua	Black & Brown	Control	-
43	5	Male (intact)	Dachshund	Brown	Control	-
44	5	Female (neutered)	Lapinkoira	Black & tan	Control	-
45	7	Female (neutered)	Mixed breed	Tri-coloured	Control	-
46	7	Female (neutered)	Belgian Shepherd Groenendael	Black	Control	-
47	4	Female (intact)	Mixed breed	Champagne	Control	-
48	7	Male (intact)	Maltese	White	Control	-
49	4	Female (intact)	King Charles Spaniel	Black & tan	Control	-
50	2	Male (intact)	Brussels Griffon	Brown	Control	-

**Table A2:** Inter-rater reliability calculated with Spearman correlation. Spearman rho is reported, with *p*-value in brackets.

Variables	Durations	Frequencies
Overall looking	0.97 [< 0.001]	0.89 [0.001]
Referential looking	0.98 [< 0.001]	0.98 [< 0.001]
Approach latency	1 [< 0.001]	-
Motor diversity	-	0.96 [< 0.001]
Task orientation	0.99 [< 0.001]	0.97 [< 0.001]
Negative valence	1 [< 0.001]	1 [< 0.001]

**Table A3:** Cox proportional hazard estimates of the time to approach the container.

Variable	N	Dogs That Approach the Container	Median Latency (Seconds)	95% CI for Latency (Upper-Lower)	Coefficient ^a^	HR ^b^	*p*
*Group*							
Control ^c^	25	23	1.80	1.40−3.00	-	-	-
AAI	20	20	2.30	1.60−4.40	−0.06	0.94	0.862
*BIS category*							
Low	14	14	2.00	1.60−4.40	−0.30	0.74	0.510
Average ^c^	19	17	2.20	1.80−5.60	-	-	-
High	12	12	2.30	1.40−null	−0.41	0.66	0.318
Wald test:	1.11, df = 3, *p* = 0.800						

^a^ Coefficient = log (relative hazard), ^b^ HR = hazard ratio, ^c^ Reference category.
